# A Weakly Supervised Gas-Path Anomaly Detection Method for Civil Aero-Engines Based on Mapping Relationship Mining of Gas-Path Parameters and Improved Density Peak Clustering

**DOI:** 10.3390/s21134526

**Published:** 2021-07-01

**Authors:** Hao Sun, Xuyun Fu, Shisheng Zhong

**Affiliations:** 1 Department of Mechanical Engineering, Harbin Institute of Technology at Weihai, Weihai 264209, China; 19s130232@stu.hit.edu.cn; 2 Department of Mechanical Engineering, Harbin Institute of Technology, Harbin 150000, China; zhongss@hit.edu.cn

**Keywords:** civil aero-engine, anomaly detection, weakly supervised, mapping relationship mining, improved density peak clustering

## Abstract

Gas-path anomalies account for more than 90% of all civil aero-engine anomalies. It is essential to develop accurate gas-path anomaly detection methods. Therefore, a weakly supervised gas-path anomaly detection method for civil aero-engines based on mapping relationship mining of gas-path parameters and improved density peak clustering is proposed. First, the encoder-decoder, composed of an attention mechanism and a long short-term memory neural network, is used to construct the mapping relationship mining model among gas-path parameters. The predicted values of gas-path parameters under the restriction of mapping relationships are obtained. The deviation degree from the original values to the predicted values is regarded as the feature. To force the extracted features to better reflect the anomalies and make full use of weakly supervised labels, a weakly supervised cross-entropy loss function under extreme class imbalance is deployed. This loss function can be combined with a simple classifier to significantly improve the feature extraction results, in which anomaly samples are more different from normal samples and do not reduce the mining precision. Finally, an anomaly detection method is deployed based on improved density peak clustering and a weakly supervised clustering parameter adjustment strategy. In the improved density peak clustering method, the local density is enhanced by K-nearest neighbors, and the clustering effect is improved by a new outlier threshold determination method and a new outlier treatment method. Through these settings, the accuracy of dividing outliers and clustering can be improved, and the influence of outliers on the clustering process reduced. By introducing weakly supervised label information and automatically iterating according to clustering and anomaly detection results to update the hyperparameter settings, a weakly supervised anomaly detection method without complex parameter adjustment processes can be implemented. The experimental results demonstrate the superiority of the proposed method.

## 1. Introduction

The civil aero-engine is the heart of aircraft, and it is highly reliable and has a low anomaly rate. However, the loss caused by civil aero-engine anomalies is unacceptable. According to incomplete statistics, more than 90% of civil aero-engine anomalies are related to gas paths. The cost of handling these anomalies accounts for 60% of the total maintenance cost of civil aircraft, and the cost of each repair can be as high as 2–3 million US dollars [1]. If an early intervention for gas-path anomalies can be made, passenger safety and the economic benefit of the airline can be guaranteed to the maximum extent. Therefore, a high-precision gas-path anomaly detection method is critical.

“Anomaly” is a general term for all faults. “Gas-path anomaly detection” refers to the determination of all anomalies without judging their specific types, while “fault diagnosis” refers to the determination of all anomalies as well as their types. The former requires fewer anomaly samples, while the latter requires more fault samples. Extensive academic research has been carried out on civil aero-engine gas-path anomaly detection and fault diagnosis. Generally, this research can be divided into two parts: studies based on the physical or mathematical model and those based on the data-driven model. The fault diagnosis method based on the physical or mathematical model is limited by the model accuracy, component characteristics, multistate nonlinear coupling, and other factors, which leads to deviation in the estimation of engine performance and fault diagnosis [2].

In the field of anomaly detection, there are many classical algorithms and frameworks. Angle-based outlier detection (ABOD) [3] assumes that angles are more stable than distances in high dimensional spaces and that a sample is an outlier if most other samples are located in similar directions. However, it is difficult to apply ABOD when anomaly samples are distributed among normal samples. K-nearest neighbor (KNN) anomaly detection [4] determines anomalies by calculating the distance from each sample to the nearest K samples. KNN cannot be applied to a dataset with uneven density. Isolation forest (I-Forest) [5] treats samples more likely to be separated as anomalies. I-Forest is sensitive to global anomalies, and it is not good at dealing with local anomalies. The histogram-based outlier score (HBOS) [6] evaluates anomalies through histograms of each dimension of samples, but it ignores the relationship between the dimensions and cannot detect relationship anomalies between the dimensions. The local outlier factor (LOF) [7] is a density-based anomaly detection method; it is good at detecting local anomalies. Stochastic outlier selection (SOS) [8] calculates anomalies using affinity matrixes, and anomalies have few affinities to other samples. SOS is difficult to use when the amount of data is too large. The locally selective combination in parallel outlier ensembles (LSCP) [9] framework aims to improve the accuracy of anomaly detection through targeted integration and selection models. When there is a crossover between anomaly samples and normal samples, the effect of the LSCP will decrease. Auto-encoder (AE) [10] determines anomalies using the reconstruction error, but sometimes the AE also reconstructs the anomalies well. The memory-augmented deep autoencoder (MenAE) [11] augments the autoencoder with a memory module, and the reconstruction thus tends to be close to normal samples. The MenAE significantly improves the accuracy of anomaly detection. In the field of weakly supervised methods, some algorithms involve a label denoising method. The graph convolutional label noise cleaner (GCN) [12] has been proposed to remove the noise in labels. Using the GCN for weakly supervised anomaly detection reduces the impact of erroneous labels. However, the input order of the features affects the convolution operation, which is not consistent with the characteristics of the gas path. A weakly supervised self-reasoning framework [13] has been proposed to improve the accuracy of video anomaly detection. It generates pseudo-labels by using binary clustering of spatiotemporal video features which helps in mitigating the noise present in the labels of anomalous videos. However, even if the noise in the label is removed, the neural network-based classifier cannot solve the extreme class imbalance of gas path parameters. The clustering assisted weakly supervised (CLAWS) method [14] uses a normalcy suppression mechanism to minimize anomaly scores of the normal regions of a video by taking into account the overall information available in one training batch. However, video anomaly detection only needs to consider the temporal dimension, not the parameter dimension. The civil aero-engine gas path has multiple parameters, and the unimportant parameters also need to be suppressed. Due to the high dimension, noise, extreme class imbalance, and other reasons, these anomaly detection methods have a poor performance in the field of gas-path anomaly detection.

The latest data-driven gas-path anomaly detection methods are mainly represented by the following kinds: (1) Mining the aero-engine baseline with principal component analysis (PCA) combined with a deep belief network (DBN) and an Elman neural network optimized by chaos particle swarm. A particle swarm and extreme learning machine have been used for further follow-up anomaly detection [15,16,17]. (2) Mining the deviation value of gas-path parameters by establishing a mathematical model and residual forward neural network. Then, anomalies are detected using a support vector machine [18,19,20]. (3) Using the original gas-path parameters from the QAR or the ACARS directly. Anomalies are detected by stacking denoising auto-encoders (SDAEs), convolutional neural networks, autoencoders combined with Gaussian models, and multisource information fusion combined with feedforward neural networks [21,22,23]. However, the baseline model is hard to establish when the data are insufficient. The mining accuracy of deviation values is still limited. The original gas-path parameters have too much noise and too many complex correlations to use. Regardless of what kind of data is used for anomaly detection, it is necessary to extract features first. The quality of feature extraction is directly related to the accuracy of anomaly detection. At the same time, the extreme class imbalance between normal samples and anomalous samples dramatically affects the accuracy of common anomaly detection methods.

With the rapid development of artificial intelligence and computer hardware, various complex algorithms have been proposed. Time-series data often contain more useful information. A recurrent neural network (RNN) and its improved algorithm are the preferred algorithms for processing time-series data. However, RNNs have a severe vanishing gradient and exploding gradient when facing excessively long time series [24]. A long short-term memory neural network (LSTM) alleviates the vanishing gradient and explodes gradient problems by controlling the long-term information that needs to be saved or forgotten through its unique gate structure [25]. In addition, a gate recurrent unit (GRU) has a similar principle to the LSTM, with fewer gate structures and easier convergence, but the GRU does not perform as well as the LSTM on large data sets [26].

As time series become increasingly longer, the LSTM and GRU are increasingly less able to learn critical information. In addition, these models cannot effectively express information in a hierarchical structure. The appearance of the attention mechanism (Attn) significantly improves this point. The attention mechanism is a particular structure embedded in machine learning models that can automatically calculate each input data’s contribution to the model, make more efficient use of useful information, and ignore useless information [27]. The self-attention mechanism is a variant of the attention mechanism, which reduces the dependence on the external model. The self-attention mechanism directly captures the internal correlation of data or features. It takes the whole time series as the observation scope to obtain the similarity of any two elements in the time series through a one-step matrix calculation [28].

However, regardless of how the neural network is optimized, the training process will be affected by the majority-class samples, resulting in the accuracy reduction of the minority class, and it has no advantage when facing extreme class imbalance. Density clustering performs well in dealing with extreme class imbalance. Density-based spatial clustering of applications with noise (DBSCAN) can cluster dense data sets of any shape, but it is susceptible to hyperparameters, and the tuning process of hyperparameters is relatively complex [29]. Ordering points to identify the clustering structure (OPTICS) improves the DBSCAN algorithm, which mainly solves sensitivity to input parameters [30]. Clustering by fast search and find of density peaks (DPC) is an effective and quick algorithm for quickly searching for clustering centers based on the local density and relative distance attributes [31]. DPC uses the decision graph to find the density peak as the clustering center and it does not need to specify the number of clusters in advance and can cluster any data shape. However, there are defects in local density and relative distance, so the DPC clustering results for complex data are not satisfactory. Additionally, there is no uniform measurement for calculating local density in the DPC, and different measurement methods are selected according to different data sets. Third, the cutoff distance only considers the global distribution of data, ignoring the local information and impacting the clustering results. Some scholars have proposed different improved DPC algorithms [32].

To solve the above problems, a weakly supervised gas-path anomaly detection method for civil aero-engines based on mapping relationship mining of gas-path parameters and improved density peak clustering is proposed. The method works by mining the complex mapping relationships of gas-path parameters using the LSTM and the attention mechanism. It then combines a classifier with a new loss function, named weakly supervised cross-entropy under extreme class imbalance, to force the mapping relationship to show the difference between normal and anomalous data. Finally, anomalies are detected using an improved density peak clustering-based anomaly detection method. In this method, weakly supervised clustering strategies are set for the engines that have multiple anomalous samples, which greatly reduces the difficulty of adjusting parameters.

In Section 2 of this paper, the complete model is introduced. In Section 3, the weakly supervised feature extraction algorithm based on mapping relationship mining is introduced. In Section 4, the weakly supervised anomaly detection algorithm based on improved density peak clustering is introduced. Finally, Section 5 introduces the experimental settings and results in detail.

## 2. Proposed Framework

Civil aero-engine gas-path components have multiple monitoring parameters, including altitude (Alt), indicated fan speed (FS), core speed (CS), total air temperature (TAT), exhaust gas temperature (EGT), fuel flow (FF), oil pressure (OP), oil temperature (OT), and Mach (Ma). These parameters of high latitudes and high redundancy are difficult to analyze without processing. There are complex mapping relations among these parameters, which can evaluate the health status of gas-path components. Traditional physical and mathematical models have difficulty expressing this mapping relationship accurately and reliably. Artificial neural networks have several advantages: (1) fully approximate arbitrary complex nonlinear relations; (2) high robustness and fault tolerance; and (3) the capability to learn and adapt to unknown systems. Therefore, the mapping relationships of gas-path parameters can be learned through a neural network, and anomalies can be found according to the deviation degree between the actual data and the predicted data of the mapping relationship.

**Figure 1:**
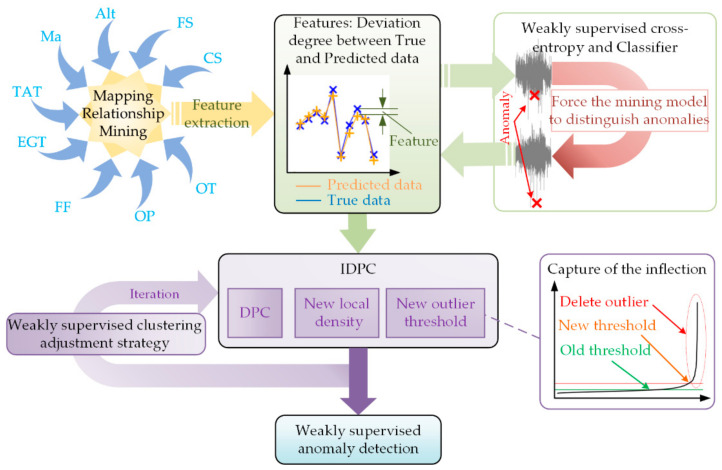
Proposed framework.

The customer notification report (CNR) provides a few anomalous labels for the gas-path parameters. Labeled data are all anomalous data, while unlabeled data are with high probability normal data. It is unknown whether the unlabeled data are anomalous. These anomaly labels can provide a guide to neural networks. Due to the extreme class imbalance between anomalous and normal samples and the small number of anomaly labels, both traditional supervised learning methods and unsupervised learning methods cannot make efficient use of label information in this situation. Therefore, a weakly supervised gas-path anomaly detection method based on mapping relationship mining of gas-path parameters and improved density peak clustering, as shown in Figure 1, is proposed in this paper. The weakly supervised loss function uses label information to improve the neural network’s effect, and improved density clustering (IDPC) overcomes the impact of class imbalance.

First, the mapping relationship between the gas-path parameters is mined by combining the LSTM with the attention mechanism. According to this mapping relationship, the theoretical value or predicted value (similar to the baseline) under specific conditions can be derived. The deviation degree between the true value and predicted value is taken as the feature (similar to the gas path deviation value). Second, there are few anomaly samples and fewer anomaly labels. The mapping relational mining model is forced to distinguish anomalous samples from normal samples using a classifier and a new loss function, named weakly supervised cross-entropy under extreme class imbalance ($Loss_{WSCE}$). Finally, clustering and anomaly detection can be achieved by adding three strategies to the DPC: (1) a local density measurement criterion based on K-nearest neighbors; (2) automatically grabbing the outlier threshold and canceling outliers’ participation in clustering; and (3) determining the K-nearest neighbor number through a weakly supervised clustering parameter adjustment strategy.

In Section 3 and Section 4, the model details are introduced. In Section 5, comparative experiments are described to demonstrate that each part of the proposed framework is reasonable. Several classical feature-extraction methods and clustering methods were used as comparative experiments to prove the superiority of the proposed framework.

## 3. Mapping Relationship Mining-Based Weakly Supervised Gas-Path Feature Extraction Method

### 3.1. Complete Feature Extraction Model

**Figure 2:**
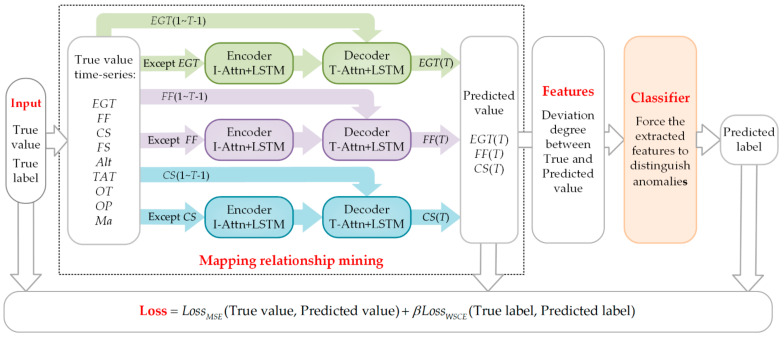
Mapping relation mining-based weakly supervised gas-path feature extraction (WSFE) method.

A weakly supervised feature extraction (WSFE) algorithm based on mapping relationship mining is proposed to extract features, as shown in Figure 2.

Exhaust gas temperature (EGT), core speed (CS), and fuel flow (FF) are three essential parameters, and many anomalies are directly reflected in them. The gas path deviation value is also based on them. Therefore, they are used as observation parameters for WSFE.

First, the original gas path parameters are divided into time series of length *T* by sliding windows. The attention mechanism is combined with the LSTM to construct encoders and decoders for mining mapping relationships. The mapping relationship from EGT, CS, and FF to others is mined. The mining model for a mapping relationship from a single parameter to other parameters is established three times to input more information and prevent the mapping relation mining accuracy from being insufficient.

To save space, the first encoder and decoder are used as examples to introduce the details of the mapping relationship mining model. To mine the mapping relationship from *EGT*(*T*) (*EGT* at time *T*) to the other parameters, the model is set as follows:

(1) The encoder is composed of an LSTM and an attention mechanism named input attention (I-Attn). It inputs a time series with length *T* for other parameters except for *EGT*. The most relevant input parameters are selected and weighted by the I-Attn and the previous encoder LSTM hidden state. This weight is conducive to the LSTM learning the deep information between parameters. The encoder outputs something learned (also called encoding).
(2) The decoder is composed of an LSTM and an attention mechanism named temporal attention (T-Attn). It inputs the encoding and the time 1 to time *T*-1 of *EGT* (also called *EGT*(1~*T*-1)). The T-Attn is used to weight different time steps of encoding, which helps the decoder LSTM to capture the long-term temporal dependencies. The decoder outputs the learned theoretical value (predicted value) of *EGT* at time *T* (*EGT*(*T*)). In this part, the mean square error loss function ($Loss_{MSE}$) is used to calculate the loss from the predicted value to the true value.
(3) The deviation degree between the true and predicted value is the extracted feature, which indicates how much the actual operation state deviates from the ideal operation state.

An over-complex classifier makes the complete model difficult to train, the class imbalance data make it difficult to classify the features, and the output (predicted labels) of any classifier can hardly be used for anomaly detection. However, by combining a simple classifier with a new loss function, named weakly supervised cross-entropy under class imbalance ($Loss_{WSCE}$), and calculating the loss of true labels and predicted labels, the mapping relationship mining model can be forced to better reflect the difference between most of the normal samples and the known anomalies, and a better feature extraction effect can be achieved.

To force the mined mapping relationship to better distinguish the anomalies, a classifier combined with $Loss_{WSCE}$ is set for feature classification. This classifier inputs the extracted features and outputs the predicted labels. The predicted labels are used only to calculate the $Loss_{WSCE}$.

The mapping relationship mining model and classifier are trained simultaneously. The outputs of the WSFE model are the extracted features. The complete loss function of it is:

(1) $$Loss=Loss_{MSE}+\beta Loss_{WSCE},$$

(2) $$Loss_{MSE}=\frac{1}{n}\sum_{i=1}^{n}{\left({\hat{y}}_{i}-y_{i}\right)}^{2},$$

where β is used to adjust the magnitude of the different loss functions to facilitate model training, $Loss_{MSE}$ is the *MSE* loss function, *y_i_* is the true value, ${\hat{y}}_{i}$ is the predicted value, and $Loss_{WSCE}$ is the weakly supervised cross-entropy loss function under extreme class imbalance, which is described in detail in Section 3.3.

The complete model training mechanism is described below:

(1) The mapping relationship mining model inputs the true values and true labels of gas-path parameters and outputs the predicted values.
(2) The deviation degree between the true and predicted values is calculated as the extracted features.
(3) The classifier inputs the extracted features and outputs the predicted labels.
(4) The $Loss_{MSE}$ is calculated by the predicted values and true values, and the $Loss_{WSCE}$ is calculated by the predicted labels and true labels. The *Loss* of the *WSFE* is calculated by the $Loss_{MSE}$ and $Loss_{WSCE}$.
(5) The complete *WSFE* model is trained by the *Loss*. At the beginning of training, the mapping relation mining precision increases rapidly, the $Loss_{MSE}$ descends rapidly, the extracted features are not accurate enough, the $Loss_{WSCE}$ fluctuates a lot, and the complete *Loss* descends rapidly. As the accuracy of the mapping relationship mining ascends to a certain degree, the descending speed of *Loss* and $Loss_{MSE}$ becomes slow, the $Loss_{WSCE}$ starts to descend significantly, and the extracted features begin to show significant differences between anomaly samples and normal samples. The training procedure is stopped until the *Loss* barely changes.

The following sections introduce the mapping relationship mining algorithm, the weakly supervised cross-entropy loss function under extreme class imbalance, and the classifier used to enhance the feature extraction effect.

### 3.2. Mapping Relationship Mining Algorithm

**Figure 3:**
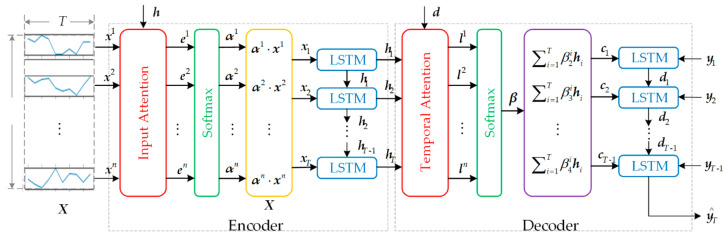
Mapping relationship mining algorithm.

The mapping relation mining algorithm adopts the seq2seq (encoder-decoder) structure, which is commonly used in machine translation [33,34], as shown in Figure 3. ***X*** = (***x***^1^, ***x***^2^,…, ***x****^n^*)^T^ = (***x***_1_, ***x***_2_,…, ***x****_T_*) represents a time series of the input data, the length of the time series is *T,* and the dimension is *n*. In the symbol of type $x_{t}^{i}$, the superscript represents the *i*th feature, and the subscript represents the data at time *t*. Words in bold italics denote matrices or vectors, and $x_{}^{T}$ denotes the transpose of vectors or matrices ***x***.

(1) Encoder

The input data are composed of multiple time series (matrix) with dimensions *n* and length *T*. In Figure 3, a time series ***X*** is taken as an example to demonstrate the mapping relationship mining process. Since the calculation of attention depends on the last moment hidden state of the LSTM, the LSTM is introduced first. For the time series ***X*** = (***x***^1^, ***x***^2^,…, ***x****^n^*)^T^ = (***x***_1_, ***x***_2_,…, ***x****_T_*), if no attention mechanism is added, then the encoder is an ordinary LSTM neural network, whose purpose is to learn a linear mapping from ***x****_t_* to ***h****_t_*:

(3) $$\begin{array}{ll} h & =(h^{1},h^{2},\ldots ,h^{n}{)}^{T}=(h_{1},h_{2},\ldots ,h_{T})\\ & =(f_{1}\left(h_{0},x_{1}\right),\;f_{1}\left(h_{1},x_{2}\right),\ldots ,f_{1}\left(h_{T-}{}_{1},x_{T}\right)), \end{array}$$

where $h_{t}$ represents the hidden state of LSTM at time *t*, and *m* represents the size of the hidden state. *f*_1_ represents the nonlinear activation function of the LSTM.

Each LSTM unit includes the forget gate, input gate, and output gate. ***f****_t_*, ***i****_t_*, and ***o****_t_* represent the expression of these three gates at time *t*, and ***s****_t_* represents the state of the memory cell at time *t*:

(4) $$f_{t}=\sigma \left(W_{f}\left[h_{t-1};x_{t}\right]+b_{f}\right),$$

(5) $$i_{t}=\sigma \left(W_{i}\left[h_{t-1};x_{t}\right]+b_{i}\right),$$

(6) $$o_{t}=\sigma \left(W_{o}\left[h_{t-1};x_{t}\right]+b_{o}\right),$$

(7) $$s_{t}=f_{t}\cdot \;s_{t-1}+i_{t}\cdot \tanh(W_{s}[h_{t-1};\;x_{t}]+b_{s}),$$

(8) $$h_{t}=o_{t}\cdot \tanh(s_{t}),$$

where $\left[h_{t-1};x_{t}\right]$ represents a concatenation of previously hidden state $h_{t-1}$ and current input $x_{t}$, $\left[h_{t-1};x_{t}\right]\in {\mathbb{R}}^{m+n}$, σ is the sigmoid activation function, $W_{f},W_{i},W_{o},W_{s}$$\in {\mathbb{R}}^{m\times (m+n)}$, and $b_{f},b_{i},b_{o},b_{s}\in {\mathbb{R}}^{m}$ are parameters to learn.

For the time series ***X*** = (***x***_1_, ***x***_2_,…, ***x****_T_*) = (***x***^1^, ***x***^2^,…, ***x****^n^*)^T^ (***X*** $\in {\mathbb{R}}^{n\times T}$), after adding input attention (I-Attn), the element $e_{t}^{k}$ in the input attention output $e_{}^{k}$ becomes:

(9) $$e_{t}^{k}=v_{e}^{T}\tanh(W_{e}[h_{t-1};\;s_{t-1}]+U_{e}x^{k}),$$

where $h_{t-1}$ and $s_{t-1}$ represent the hidden state and the memory cell of the LSTM at time *t*−1. $v_{e}\in {\mathbb{R}}^{T}$, $W_{e}\in {\mathbb{R}}^{T\times 2m}$, and $U_{e}\in {\mathbb{R}}^{T\times T}$ are parameters to learn.

Through the SoftMax layer, the element $\alpha_{t}^{k}$ of output $\alpha_{}^{k}$ becomes:

(10) $$\alpha_{t}^{k}=\frac{\exp(e_{t}^{k})}{\sum_{i=1}^{n}\exp(e_{t}^{i})},$$

where $\alpha_{t}^{k}$ is the input-attention weight of the *k*-th feature at time *t*, and the weighted sum of all inputs is guaranteed to be 1 through the SoftMax layer.

Then, by adding input attention, the input becomes:

(11) $$\tilde{X}={\left(\alpha^{1}x^{1},\alpha^{2}x^{2},\ldots ,\alpha^{n}x^{n}\right)}^{T},$$

Input $\tilde{X}$ into the LSTM to update the encoder’s learning result:

(12) $$\begin{array}{ll} h & =(h^{1},h^{2},\ldots ,h^{n}{)}^{T}=(h_{1},h_{2},\ldots ,h_{T})\\ & =(f_{1}\left(h_{0},{\tilde{x}}_{1}\right),\;f_{1}\left(h_{1},{\tilde{x}}_{2}\right),\ldots ,f_{1}\left(h_{T-}{}_{1},{\tilde{x}}_{T}\right)) \end{array}$$

where *f*_1_ represents the nonlinear activation function of the LSTM unit, which can be obtained from Equations (4)–(8). The above settings add weights to the input data instead of equally treating all the inputs, improving the model effect and convergence speed.

(2) Decoder

Similarly, the weight ***l****_t_* of temporal attention (T-Attn) at time *t* depends on the hidden state *d_t_*_−1_ and memory cell state ${s^{\prime }}_{t-1}^{}$ of the LSTM at time *t*−1, and the elements $l_{t}^{i}$ of temporal attention weight ***l****_t_* at time *t* are:

(13) $$l_{t}^{i}=v_{d}^{T}\tanh(W_{d}[d_{t-1};\;{s^{\prime }}_{t-1}]+U_{d}h_{i}),1\leq i\leq T,$$

where $d_{t-1}\in {\mathbb{R}}^{p}$ and ${s^{\prime }}_{t-1}\in {\mathbb{R}}^{p}$ represent the hidden state and memory cell of LSTM at time *t*‒1, respectively, and $[d_{t-1};\;{s^{\prime }}_{t-1}]\in {\mathbb{R}}^{2p}$ represents a concatenation of $d_{t-1}$ and ${s^{\prime }}_{t-1}$. *p* represents the size of the hidden state. $v_{d}\in {\mathbb{R}}^{m}$, $W_{d}\in {\mathbb{R}}^{m\times 2p}$, and $U_{d}\in {\mathbb{R}}^{m\times m}$ are parameters to learn.

Through the SoftMax layer, the elements $\beta_{t}^{i}$ in ***β****_t_* become:

(14) $$\beta_{t}^{i}=\frac{\exp(l_{t}^{i})}{\sum_{j=1}^{n}\exp(l_{t}^{j})},$$

where the attention weight $\beta_{t}^{i}$ represents the importance of the *i*th encoder hidden state at time *t*.

The weighted sum of all the encoder hidden states $\left(h_{1},h_{2},\ldots ,h_{T}\right)$ is the context vector c, and the element in ***c*** is:

(15) $$c_{t}^{}=\sum_{i=1}^{T}\beta_{t}^{i}h_{i},$$

Combine ***c****_t_* with the decoder input $y={\left(y_{1},y_{2},\ldots ,y_{T-1}\right)}^{T}\in {\mathbb{R}}^{\left(T-1\right)}$ to obtain $\tilde{y}={\left({\tilde{y}}_{1},{\tilde{y}}_{2},\ldots ,{\tilde{y}}_{T-1}\right)}^{T}$ $\in {\mathbb{R}}^{\left(T-1\right)}$, and the elements ${\tilde{y}}_{t-1}$ in $\overset{\sim }{y}$ are:

(16) $${\tilde{y}}_{t-1}={\tilde{w}}^{T}[y_{t-1},c_{t-1}]+\tilde{b},$$

$\tilde{w}\in {\mathbb{R}}^{m+1}$ and $\tilde{b}\in \mathbb{R}$ map the concatenation matrix $\left[y_{t-1},c_{t-1}\right]$$\in {\mathbb{R}}^{m+1}$ to the size of the decoder input. Use $\tilde{y}={\left({\tilde{y}}_{1},{\tilde{y}}_{2},\ldots ,{\tilde{y}}_{T-1}\right)}^{T}$ to update the hidden state of the LSTM:

(17) $$\begin{array}{ll} d & =(d_{1},d_{2},\ldots ,d_{T})\\ & =\;\left(f_{2}\left(d_{0},{\tilde{y}}_{0}\right),\;f_{2}\left(d_{1},{\tilde{y}}_{1}\right),\ldots ,f_{2}\left(d_{T-1},{\tilde{y}}_{T-1}\right)\right) \end{array}$$

Similarly, the nonlinear function *f*_2_ can be calculated from Equations (4)–(8):

(18) $$f_{t}^{\prime }=\sigma \left(W_{f}^{\prime }\left[d_{t-1};{\tilde{y}}_{t-1}\right]+b_{f}^{\prime }\right),$$

(19) $$i_{t}^{\prime }=\sigma \left(W_{i}^{\prime }\left[d_{t-1};{\tilde{y}}_{t-1}\right]+b_{i}^{\prime }\right),$$

(20) $$o_{t}^{\prime }=\sigma \left(W_{o}^{\prime }\left[d_{t-1};{\tilde{y}}_{t-1}\right]+b_{o}^{\prime }\right),$$

(21) $$s_{t}^{\prime }=f_{t}^{\prime }\cdot \;s_{t-1}^{\prime }+i_{t}^{\prime }\cdot \tanh(W_{s}^{\prime }[d_{t-1};{\tilde{y}}_{t-1}]+b_{s}^{\prime }),$$

(22) $$d_{t}=o_{t}^{\prime }\cdot \tanh(s_{t}^{\prime }),$$

where $[d_{t-1};{\tilde{y}}_{t-1}]\in {\mathbb{R}}^{p+1}$ is a concatenation matrix of previous hidden state $d_{t-1}$ and the decoder input ${\tilde{y}}_{t-1}$. $W_{f}^{\prime },W_{i}^{\prime },W_{o}^{\prime },W_{s}^{\prime }$ $\in {\mathbb{R}}^{p\times (p+1)}$ and $b_{f}^{\prime },b_{i}^{\prime },b_{o}^{\prime },b_{s}^{\prime }\in {\mathbb{R}}^{p}$ are the parameters to learn. σ is the sigmoid activation function.

Therefore, the predicted value ${\hat{y}}_{T}$ at time *T* can be expressed by:

(23) $$\begin{array}{ll} {\hat{y}}_{T} & =F\left(y_{1},y_{2},\ldots ,y_{T-1},x_{1},x_{2},\ldots ,x_{T}\right)\\ & =v_{y}^{T}\left(W_{y}\left[d_{T},c_{T}\right]+b_{w}\right)+b_{v} \end{array}$$

where $[d_{T};c_{T}]\in {\mathbb{R}}^{p+m}$ is a concatenation matrix of the decoder hidden state $d_{T}$ and the context vector $c_{T}$. $W_{y}\in {\mathbb{R}}^{p\times (p+m)}$ and $b_{w}\in {\mathbb{R}}^{p}$ map $\left[d_{T};c_{T}\right]$ to the size of $d_{T}$. $v_{y}\in {\mathbb{R}}^{p}$ and $b_{v}\in \mathbb{R}$ are parameters to learn, which produces the result.

### 3.3. Weakly Supervised Cross-Entropy Loss Function under Extreme Class Imbalance

The cross-entropy loss function (CE) [35] can be used for the classification task, but CE does not perform well in the class imbalance dataset. Focal loss (FL) [36] can force the model to focus on minority classes in the class-imbalanced dataset. However, these two loss functions only apply to supervised learning and cannot be used when there are not enough labels. To extract features under extreme class imbalance and take advantage of weakly supervised labels, new loss functions based on CE and FL that can use weakly supervised labels must be investigated.

Therefore, a weakly supervised cross-entropy loss function ($Loss_{WSCE}$) under extreme class imbalance is proposed:

(24) $$Loss_{WSCE}=-y\log(P)-\alpha (1-y)P^{\gamma }\log(1-P),$$

where *y* represents the label, with *y* = 0 when the sample is anomalous and *y* = 1 when the sample is not a known anomaly. *P* represents the classifier’s output which is also the probability that the sample is normal. *α* represents an imbalanced coefficient equal to the ratio of known anomalies to total samples. *γ* represents a focusing coefficient which is used to adjust the loss descent speed, *γ* ≥ 0.

When the sample is a known anomaly (*y* = 0), the closer the output *P* is to 0, the smaller the $Loss_{WSCE}$ will be. When the sample is not a known anomaly (*y* = 1), the closer the output *P* is to 1, the smaller the $Loss_{WSCE}$ will be. The imbalanced coefficient *α* is used to adjust the weight of −ylog(P) and $\left(1-y)P^{\gamma }\log(1-P\right)$. It can force the model to pay more attention to the known anomaly samples, and prevent the model training procedure from being dominated by a large number of normal samples. The focusing coefficient *γ* is used to adjust the descent speed of $Loss_{WSCE}$. When the descent speed of $Loss_{WSCE}$ is similar to that of $Loss_{MSE}$, the difficulty of the model training will be reduced.

### 3.4. Classifier for Enhancing Feature Extraction Effect

Due to the inseparability of extracted features and extreme class imbalance, a simple classifier’s ability to identify anomalies is minimal. Classifiers with complex structures are difficult to train, and they show little improvement in classification. The simple classifier combined with the $Loss_{WSCE}$ can effectively improve the feature extraction effect, and there are apparent differences between normal and anomalous samples in the extracted features.

**Figure 4:**
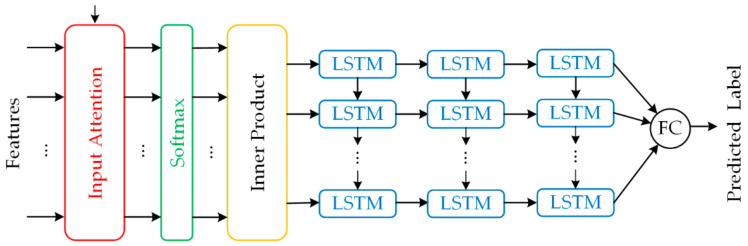
Classifier.

The classifier structure is shown in Figure 4. It adopts the same structure as the encoder model and is followed by two additional LSTM layers and a fully connected layer. To save space, the formula derivation of the classifier is not introduced. The number of fully connected (FC) layer units is 1, the activation function is sigmoid, and the loss function is $Loss_{WSCE}$, which calculates the loss of the predicted label and the true weakly supervised label.

## 4. Improved Density Peak Clustering-Based Anomaly Detection Method

Clustering using the fast search and find of density peaks (DPC) algorithm assumes that cluster centers are characterized by a higher density than their neighbors and by a relatively large distance from points with higher densities. The DPC algorithm proposes three concepts: for each sample *i*, the local density $\rho_{i}$ of it, the cutoff distance $d_{c}$, and the distance $\delta_{j}$ from points of higher density to sample *i*:

(25) $$\rho_{i}=\sum_{j}\chi (d_{ij}-d_{c}),$$

(26) $$\chi (d_{ij}-d_{c})=\left\{\begin{array}{c} 1,\;x<0\\ 0,\;x\geq 0 \end{array}\right.,$$

(27) $$\delta_{j}=\min_{j:\rho_{j}>\rho_{i}}(d_{ij}),$$

where $d_{ij}$ is the Euclidean distance of sample *i* and sample *j*. The cutoff distance $d_{c}$ is a hyperparameter, which can be adjusted according to the clustering effect.

For the anomaly detection task in this study, different engines were considered have different health states, so the extracted features inevitably reduce the accuracy when they participate in density clustering together. However, a single engine dataset is small, and the local density is greatly affected by the cutoff distance. The DPC divides the clustering center using a decision graph and classifies sample *j* (which is not the clustering center) to the closest center with a larger density than sample *j*. Once a clustering division error is made, it easily leads to the remaining points’ linkage error. For this reason, many scholars have proposed local density definition methods using the K-nearest neighbors and new sample distribution strategies.

The purpose of the existing improved DPC algorithm is clustering rather than anomaly detection. Outliers may be divided into clustering centers or assigned to the nearest class. The outliers described in this paper are usually independent of all classes, and the correlation between outliers is tiny. Rudely treating them as clustering centers or assigning them to other classes would bring significant errors to the anomaly detection task. The number of K-nearest neighbors needs to be adjusted several times, which is highly subjective. Therefore, an anomaly detection method based on improved density peak clustering (IDPC) and a weakly supervised clustering adjustment strategy is proposed. It can improve the anomaly detection accuracy without being affected by class imbalance.

### 4.1. Anomaly Detection Method Base on Improved DPC

The authors of a previous study [32] proposed new definitions of local density and outliers. The exponential kernel with width δ=1 defines the local density:

(28) $$\rho_{i}=\sum_{j\in KNN(i)}\exp(-d_{ij}),$$

where $d_{ij}$ is the Euclidean distance of sample *i* and sample *j*, and *KNN*(*i*) is the set of K-nearest neighbors of sample *i.* The outlier threshold is defined by:

(29) $$k_{dist}(i)=\max_{j\in KNN(i)}\{d_{ij}\},$$

(30) $$threshold=\frac{1}{N}\sum_{i=1}^{N}k_{dist}(i),$$

(31) $$Outlier=\{o|k_{dist}(o)>threshold\},$$

where $k_{dist}(i)$ is the furthest K-nearest neighbor of sample *i*.

This method finally assigns outliers to the nearest cluster after the clustering of other points is completed. Outlier thresholds are determined by averaging, resulting in a vast number of outliers selected, and the outliers may not be judged as anomalies.

**Figure 5:**
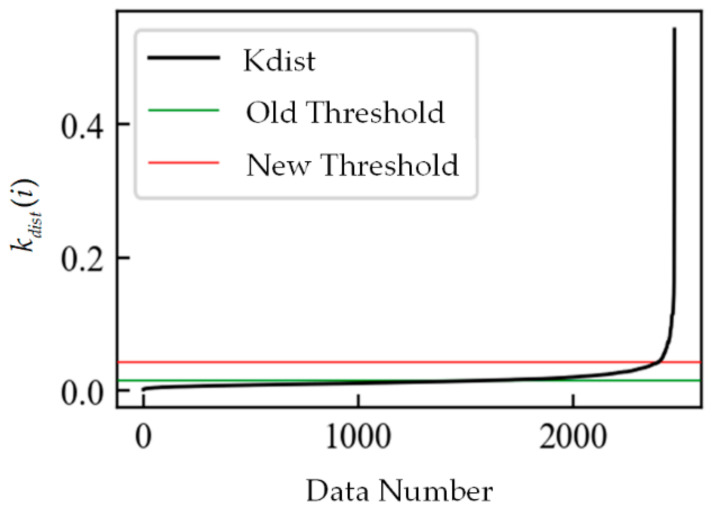
Outlier threshold.

As shown in Figure 5, the green line is the old outlier threshold, and Kdist is the curve composed of all *k_dist_*(*i*) ordered by magnitude. This old outlier threshold definition cannot accurately find the inflection point of Kdist, so a new outlier threshold definition is proposed, as shown by the red line in Figure 5.

**Figure 6:**
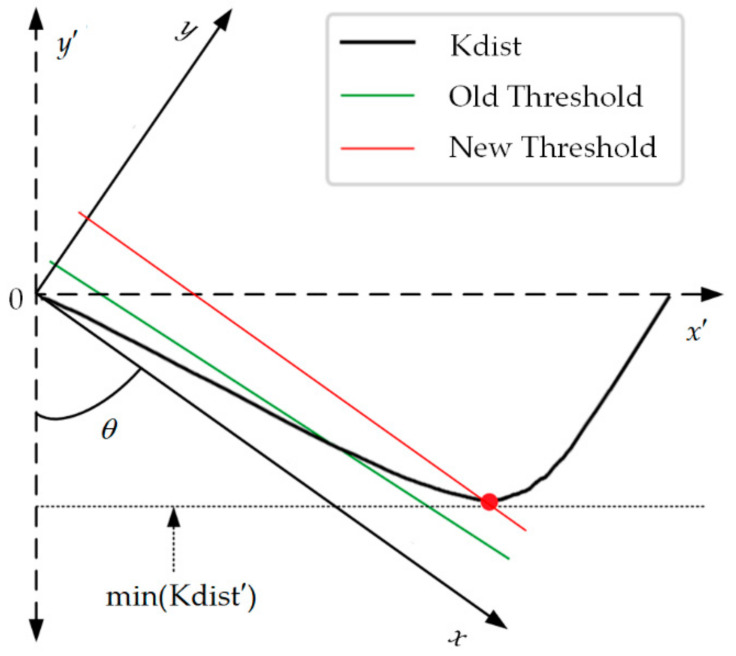
New definition of outlier threshold.

Figure 6 shows the calculation method of the new outlier threshold. The minimum value of curve Kdist is taken as the origin, and the curve Kdist is rotated to the angle shown in the figure to obtain a new coordinate axis and a new curve ${\mathrm{Kdist}}^{\prime }$. The minimum of the new curve on the new coordinate axis is the new outlier threshold, and then the corresponding position on the original coordinate axis can be determined. The coordinate transformation process is shown in Equations (32)–(34).

(32) $$\theta =\arctan\left(\frac{\max(x_{\mathrm{Kdist}})-\min(x_{\mathrm{Kdist}})}{\max(y_{\mathrm{Kdist}})-\min(y_{\mathrm{Kdist}})}\right),$$

(33) $$M_{trans}=\left[\begin{array}{cc} \cos\theta & -\sin\theta \\ \sin\theta & \cos\theta \end{array}\right],$$

(34) $$(x_{\mathrm{Kdist}}{}^{\prime },y_{\mathrm{Kdist}}{}^{\prime })=(x_{\mathrm{Kdist}}-\min(x_{\mathrm{Kdist}}),y_{\mathrm{Kdist}}-\min(y_{\mathrm{Kdist}}))\cdot M_{trans},$$

where $x_{\mathrm{Kdist}}$ and $y_{\mathrm{Kdist}}$ are vectors of all the values of Kdist along the *x*-axis and *y*-axis, $M_{trans}$ is a transformation matrix used to rotate the coordinate axes, and $x_{\mathrm{Kdist}}{}^{\prime }$ and $y_{\mathrm{Kdist}}{}^{\prime }$ are vectors of all the values of ${\mathrm{Kdist}}^{\prime }$ along the *x*′-axis and *y*′-axis.

After the coordinate transformation, the new threshold ${\mathrm{Threshold}}^{\prime }$($x_{\mathrm{Kdist}}{}^{\prime }(i)$,$\min(y_{\mathrm{Kdist}}{}^{\prime })$) can be found. The *i* of $x_{\mathrm{Kdist}}{}^{\prime }(i)$ is the index of the *i*th value in order, and then $x_{\mathrm{Kdist}}(i)$ and its corresponding outlier threshold can be determined according to the index *i*. Outliers are directly divided into anomaly classes and do not participate in the subsequent clustering process.

The complete clustering process is as follows:

(1) Calculate the local density and determine the density peak, Kdist, and outliers. Delete outliers from the clustering process. Taking *ρ* as the *x*-axis and *δ* as the *y*-axis, draw the decision graph. Determine the clustering center through the decision graph; *ρ* and *δ* of the cluster center are relatively large. Add the class label m (m = 0, 1, 2, 3, …, M) to the cluster center in order.
(2) Constantly search for the unclustered points in the K-nearest neighbors of clustering centers and divide them into the cluster of clustering centers.
(3) Calculate the probability that the remaining points belong to each class and assign them to the classes with the highest probability.

The similarity of sample *i* and each class is calculated, as shown in Equations (35) and (36), and then sample *i* is assigned to the class with the highest probability.

(35) $$s_{ij}=\left\{\begin{array}{c} \frac{1}{d_{ij}},\;d_{ij}\neq 0\\ 1,\;d_{ij}=0 \end{array}\right.,$$

(36) $$P_{i}^{m}=\sum_{y_{j}=m}^{j\in KNN(i)}w_{ij}\times s_{ij},$$

where $d_{ij}$ is the Euclidean distance of sample *i* and sample *j*; *KNN*(*i*) is the set of K-nearest neighbors of sample *I*; $s_{ij}$ is the similarity of samples *i* and *j*; $P_{i}^{m}$ is the probability that sample *i* belongs to class *m*; $w_{ij}$ is a similar weight of samples *i* and *j*, which is related to the distribution of sample *j* and the distribution of K-nearest neighbors of sample *i*, $w_{ij}=s_{ij}/(\sum_{l\in KNN(i)}s_{ij})$; and $y_{j}=m$ (*m* = 1, 2, 3,…, *M*) is the class label.

After clustering, outliers and minority classes are regarded as anomalies.

### 4.2. Weakly Supervised Clustering Parameter Adjustment Strategy

The Calinski–Harabasz (CH) index, the silhouette coefficient (SC), the Davies–Bouldin index (DBI), and the Dunn Validity index (DVI) [37,38,39,40] are unsupervised indicators for evaluating clustering. In general, the best clustering result is obtained when it has small compactness within any class and a large separation between classes. The CH index calculates the compactness and separation using the sum of squares of the sample distances. The SC evaluates compactness and separation using the obvious degree of class silhouettes. The DBI calculates the maximum ratio between the mean of the sample distance within a class and the distance from the cluster center. The DVI calculates the ratio of the shortest distance between samples of different classes to the maximum sample distance in a class.

**Figure 7:**
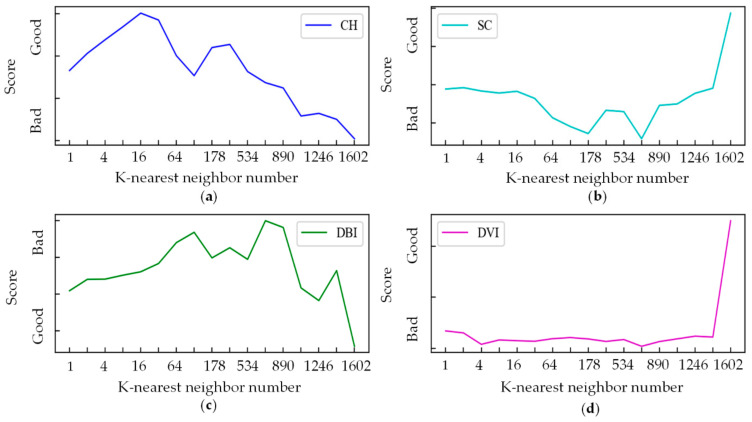
The influence of the K-nearest neighbor number on clustering. (**a**) CH index score; (**b**) SC score; (**c**) DBI score; (**d**) DVI score.

Figure 7 shows four index scores when the number of K-nearest neighbors increases from 1 to 1602 (0.9 times the total number of samples). When the number of K-nearest neighbors is large, there are few classes in the clustering results, and when the number of K-nearest neighbors is small, there are a great many classes in the clustering results. The larger the number of K-nearest neighbors, the larger the number of samples in a majority class and the fewer the classes in the clustering results, leading to the increase of the compactness and the decrease of the separation calculated by the sum of squares of the sample distances. Therefore, the CH index becomes worse when the number of K-nearest neighbors is larger. The other indicators use the mean or maximum value rather than the sum of squares to calculate the compactness and separation, avoiding the influence caused by too many samples in a majority class, so they get similar score trends.

From the perspective of unsupervised learning, for the anomaly detection task in this paper, the clustering effect is the best when the K-nearest neighbor number is large, and the clustering effect is better when the K-nearest neighbor number is small. Therefore, the K-nearest neighbor number should be large or small.

In this paper, most of the data are normal samples with a concentrated distribution. The optimal clustering result is to cluster the data into two classes: normal and anomaly. When the K-nearest neighbor number is relatively large, the clustering result is affected by the whole dataset and is closer to the ideal condition, with fewer outliers and fewer samples within minority classes. When the K-nearest neighbor number is small, the local density can better reflect the samples’ local conditions and cluster more classes, more outliers, and more samples within majority classes. Therefore, the K-nearest neighbor number should be large or small.

To use the key information contained in the labels and reduce the parameter adjustment difficulty, Alrgoithm 1 shows the pseudocode of weakly supervised clustering adjustment strategy, where *n* is the number of total samples and int(*n*) is the integer of *n*.
**Algorithm 1.** Weakly supervised clustering adjustment strategy**Input**: Features**Output**: Anomalies1 Randomly delete some anomaly labels2 Iteration range *r* = array [int (0.9 *n*), int (0.8*n*), int (0.7*n*),…, int (0.1*n*), int (0.05*n*), int(0.025*n*),…, 1]3 Sample number of minority class *n_mc_* = int (0.01*n*)4 **while** no known anomalies detected **do**5    **for** *i* in range(*r*) **do**6        *k* = *r*[i]7        Run improved DPC to cluster and find outliers8        Find minority classes9        **if** known anomalies in minority classes or outliers **then**10           **end while**11    **if** *n_mc_* ≤ int (0.1*n*)**:**12        *n_mc_* = *n_mc_* + int (0.01*n*)13    **else** anomaly detection failed14 **end while**

(1) Randomly delete some anomaly labels.
(2) The K-nearest neighbor number starts from int (0.9*n*) and decreases with int (0.1*n*). When the K-nearest neighbor number is less than int (0.1*n*), it is continuously halved and rounded until the K-nearest neighbor number is 1.
(3) Iterate continuously to divide outliers and minority classes. The minority classes are judged according to whether the sample number in this class is less than int (0.01*n*).
(4) Determine whether known anomalies are found in outliers or minority classes. If anomalies can be detected, stop the iteration.
(5) If the K-nearest neighbor number decreases to 1 and no anomaly can still be detected, *n_mc_* increases by 0.01*n*;
(6) If *n_mc_* is increased to 0.1*n* and unable to find anomalies, the anomaly detection algorithm will be considered invalid.
(7) After anomaly detection, save weakly supervised clustering parameters. Determine whether the known anomalies without labels can be detected by the algorithm. If all of them can be detected, the algorithm has the best effect; if some of them can be detected, the algorithm has a good effect; if not, the algorithm is invalid.

For engines with only one anomaly or no anomalies, the weakly supervised clustering adjustment strategy is not applicable. The K-nearest neighbor number of IDPC is constantly adjusted according to the four evaluation indexes mentioned above, and the clustering with the highest score is used for anomaly detection. However, due to the inconsistent evaluation criteria of the four indicators, the K-nearest neighbor is not easy to adjust, and due to the lack of label information, it is difficult to control the false alarm and false negatives in anomaly detection.

### 4.3. Anomaly Detection Evaluation Index

The false alarm rate and receiver operating characteristic curve (ROC) are usually used to evaluate the effect of an anomaly detection algorithm. The former is the ratio of the number of false alarms to the number of detected anomalies, while the latter is calculated based on false alarms and false negatives. The false alarm rate needs to have a sufficient number of anomalous samples, and the ROC needs to have a sufficient number of anomalous samples and fully supervised labels.

For the data used in this study, there were more than 31,000 samples in total and only 17 anomaly samples with anomaly labels. Neither the false alarm rate nor the ROC could be used to evaluate the quality of the method in this study. Considering that engines with different data sizes have different tolerances to false alarms, the effect of anomaly detection is evaluated in the following ways.

If the known anomalies can be detected by a certain method, this method is valid. The anomaly detection evaluation index is the ratio of the total number of false alarms to the total size of the dataset. The smaller this ratio is, the more accurate the anomaly detection is, and the fewer false alarms there will be.

If the known anomalies cannot be detected by a certain method, this method is invalid.

## 5. Experiments

This section describes the use of a private dataset provided by airlines for experiments. In this dataset, 31,190 pieces of data from 12 CFM56-5B2/3 engines were used for application verification. There were 17 known anomaly labels in this dataset, including 13 anomaly labels provided by the CNR and 4 anomaly labels provided by the maintenance record; the states of the other data were unknown, but they had a high probability of being normal; different engines had different health statuses and parameter fluctuation ranges. The total numbers of anomalies for each engine are shown in Table 1.

The experimental data included altitude (Alt), indicated fan speed (FS), core speed (CS), total air temperature (TAT), exhaust gas temperature (EGT), fuel flow (FF), oil pressure (OP), oil temperature (OT), and Mach (Ma). Some of the data are shown in Table 2. To prevent dimensionality from affecting model training, the Z-score standardization method was used to convert the original data into standardized data with a mean of 0 and a variance of 1, as shown in Equation (37).

(37) $$y_{i}=\frac{x_{i}-\bar{x}}{s},$$

where $y_{i}$ is the standardized data, $x_{i}$ is the original data, $\bar{x}$ is the mean of $x_{i}$, and s is the standard deviation of $x_{i}$.

To provide as much detail as possible on the model in this study, Section 5.1 explains the sample training strategy and hyperparameter setting. Section 5.2 and Section 5.4 show the feature extraction and anomaly detection results, respectively. Several comparative experiments are described in Section 5.3 and Section 5.5 to prove the superiority of the method proposed in this paper. To save space, the results of some inferior comparative experiments are not presented in detail.

### 5.1. Training Strategy and Setting of Hyperparameters

The health conditions of the engines used in the experiments were different. Some engines were new, and some engines were old engines that had been running for more than ten years. The experimental results show that the feature extraction model trained with new engines was more sensitive to anomalies, but the generalization ability for old engines was weak. When using the old engines to train the model, the generalization ability was strong, but it was not sensitive to the anomaly. Therefore, three model training strategies were proposed: (1) training the model with new engine data; (2) training the model with old engine data; (3) training the model with mixed engine data. The old engines used the WSFE model trained with new engine data to make the anomalies more apparent, while the other engines used the WSFE model trained with mixed engine data, which led to better anomaly detection results.

The above training strategy had to be done because of the limited data in this study. In theory, there is no need to use this strategy when there is sufficient data.

The hyperparameters are shown in Table 3.

### 5.2. Feature Extraction Results

**Figure 8:**
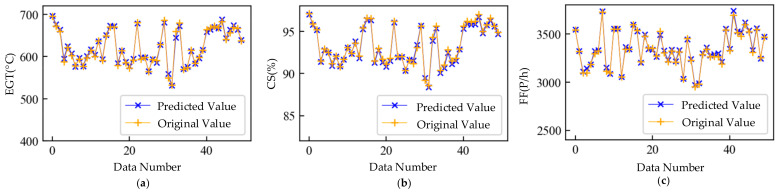
Result of mapping relation mining trained with mixed data. (**a**) Predicted and original values of exhaust gas temperature; (**b**) predicted and original values of core speed; (**c**) predicted and original values of fuel flow.

To demonstrate the mapping relationship mining results, we arranged all engine data in engine number order. The first 70% of this dataset was used as the training set to train the model, and the last 30% of this dataset was used as the verification set to avoid overfitting. In particular, there were too few anomaly samples in the dataset, and the purpose of this study was to extract the features of the whole dataset; therefore, only the validation set was used to avoid overfitting, and the test set was not divided. Figure 8 shows a comparison between some of the validation set original values and the validation set predicted values obtained by the WSFE. The blue line is the predicted value, and the yellow line is the original value. The method proposed in this paper could effectively mine the mapping relationship between the gas-path parameters, and the predicted value of the model could accurately grasp the characteristics of the original value.

**Figure 9:**
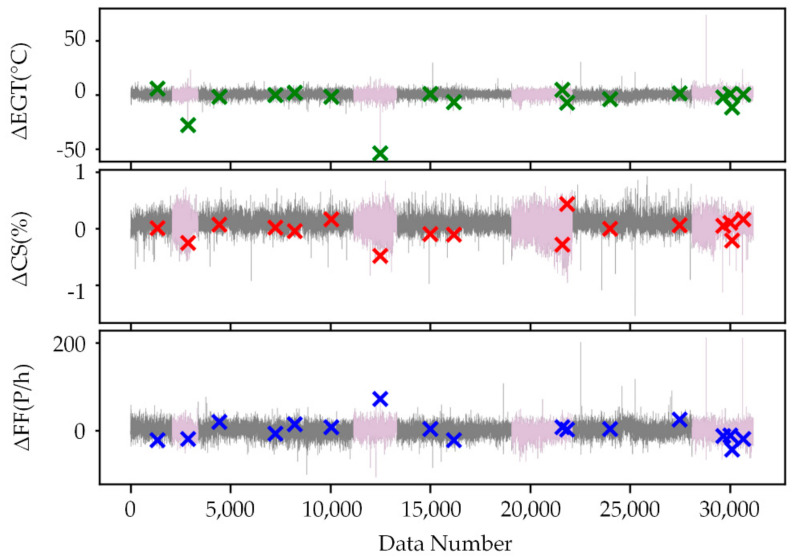
Extracted features trained with mixed engine data.

**Figure 10:**
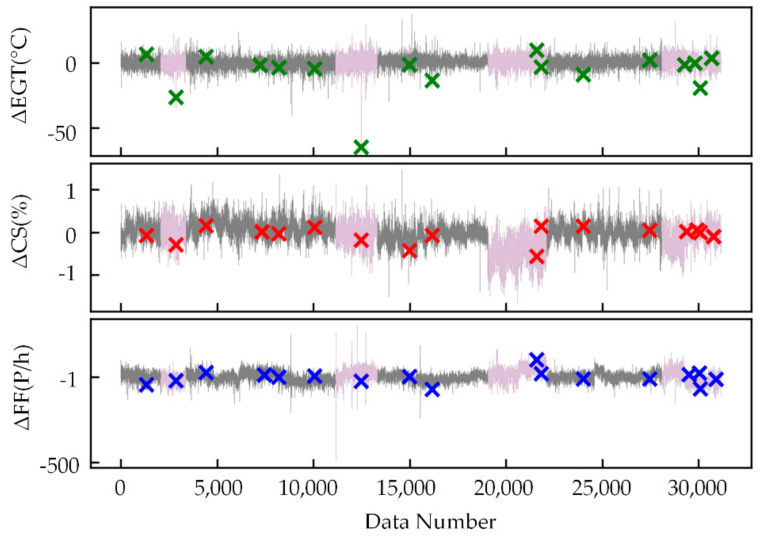
Extracted features trained with new engine data.

**Figure 11:**
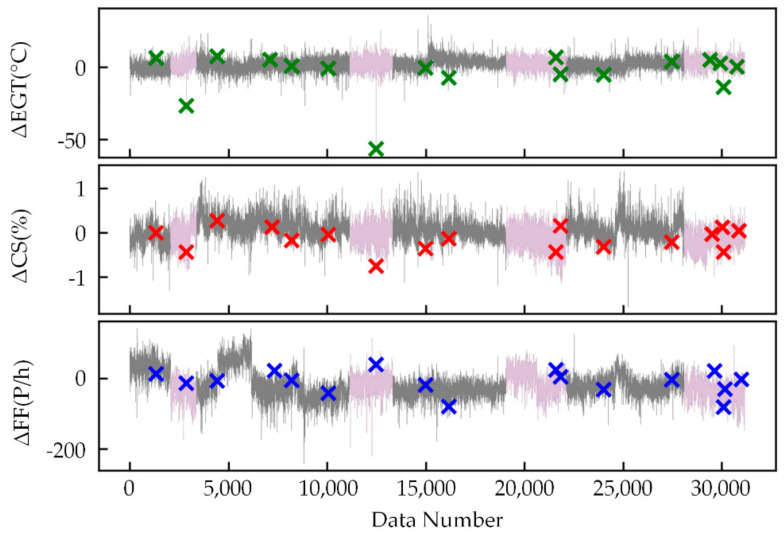
Extracted features trained with old engine data.

As shown in Figure 9, Figure 10 and Figure 11, the feature extraction results of different model training strategies using mixed engine data, new engine data, and old engine data are presented. In these figures, the *x*-axis represents the data number. The *y*-axis represents ΔEGT, ΔCS, and ΔFF, which are the deviation degrees between the original values and the predicted values of EGT, CS, and FF. The points marked as “×” are the known outliers given by CNR. The pink line is the old engine, and the gray line is the new engine. The first 70% of Figure 9 is the training set, while the last 30% is the verification set. The gray line of Figure 10 is the training set, while the pink line is the verification set. The pink line of Figure 11 is the training set, while the gray line is the verification set.

The feature extraction result using mixed data could clearly show most of the anomalies. The extracted features could obviously reflect the different health conditions of the new and old engines and the variation trend in engine performance. The new engine extraction results fluctuated less, while the old engine extraction results fluctuated more. The model trained with new engines was more sensitive to anomalies but had a poor generalization ability. The model trained with old engines had lower accuracy of mining mapping relationships but had a strong generalization ability. This made the anomalies more apparent than in the old engines that used the WSFE model trained with new engine data, and it led to better anomaly detection results.

The root mean square error (*RMSE*), mean absolute error (*MAE*), mean absolute percentage error (*MAPE*), and *R*^2^ score (*R*^2^) were used to evaluate the mining mapping relationship effect:

(38) $$RMSE=\sqrt{\frac{1}{n}\sum_{i=1}^{n}{\left({\hat{y}}_{i}-y_{i}\right)}^{2}},$$

(39) $$MAE=\frac{1}{n}\sum_{i=1}^{n}\left|{\hat{y}}_{i}-y_{i}\right|,$$

(40) $$MAPE=\frac{100\%}{n}\sum_{i=1}^{n}\left|\frac{{\hat{y}}_{i}-y_{i}}{y_{i}}\right|,$$

(41) $$R^{2}=1-\frac{RMSE}{Var},$$

where *Var* is the variance of *y*. The smaller the *RMSE*, *MAE*, and *MAPE* are, the better the mapping relationship mining is. The closer *R*^2^ is to 1, the better the mapping relationship mining is.

Table 4 shows the scores under different training methods. The mapping relationship mining model trained with the mixed engine data was the best, but whether it was the best for anomaly detection needs to be determined through the experiment in Section 5.4.

### 5.3. Comparative Experiments of Feature Extraction

This section describes the use of some different methods for comparative experiments. To save space, the results of different training methods are not presented in detail.

**Figure 12:**
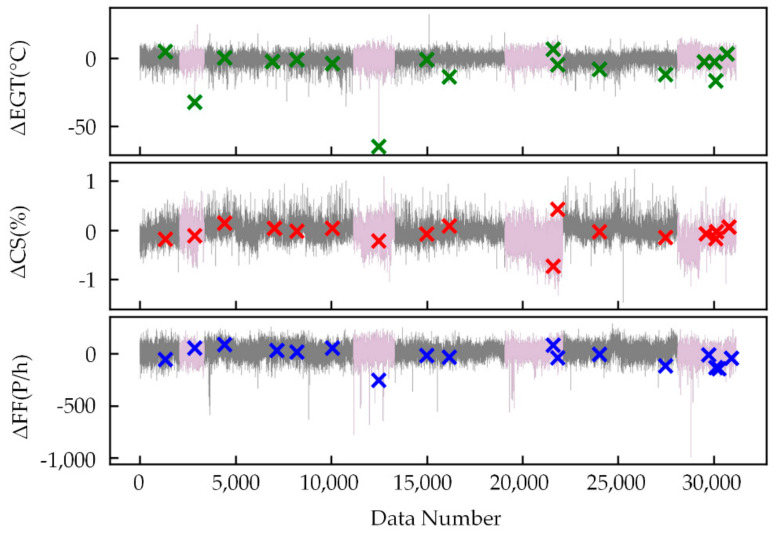
Feature extraction result without $Loss_{WSCE}$ and classifier.

Figure 12 shows the feature extraction results without $Loss_{WSCE}$ and the classifier, trained with mixed engine data. The *x*-axis is the data number, and the *y*-axis is the deviation degree between the original values and the predicted values. Points labeled “×” are known to be anomalous. The pink line is the old engine, and the gray line is the new engine. The first 70% of Figure 12 is the training set, while the last 30% is the verification set. The feature extraction effect is slightly worse than the method proposed in this paper, and some anomalies are not apparent, which proves the superiority of the loss function proposed in this paper.

Table 5 shows the scores of the feature extraction model without $Loss_{WSCE}$ and the classifier. $Loss_{WSCE}$ and the classifier hardly affect mapping relationship mining. However, $Loss_{WSCE}$ and the classifier can effectively improve the resolution of the ability to know anomalies. Comparisons of anomaly detection results are given in Section 5.5.

**Figure 13:**
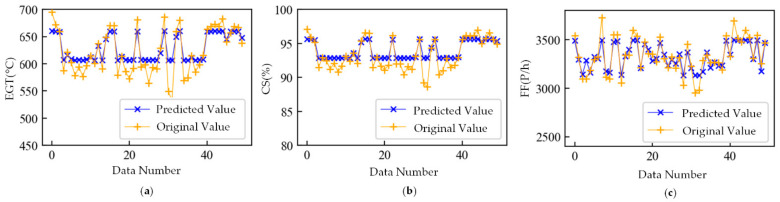
Mining results of the mapping relationship mining model with only the encoder-decoder and LSTM. (**a**) The predicted and original values of exhaust gas temperature; (**b**) the predicted and original values of core speed; (**c**) the predicted and original values of fuel flow.

Figure 13 shows the mining results of the mapping relationship mining model with only the encoder-decoder and the LSTM, which is seriously overfitted and can hardly learn the correct mapping relationship of gas-path parameters. This model cannot mine the deep features of gas-path parameters, and it is an invalid model, which proves the superiority of the model structure proposed in this paper.

**Figure 14:**
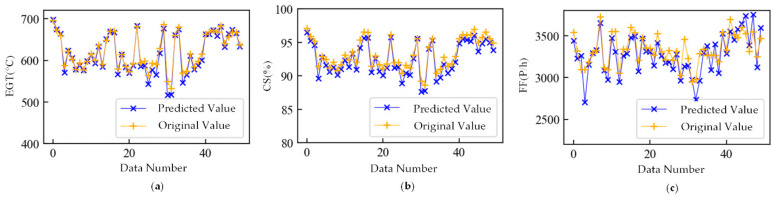
Mining results of the mapping relationship mining model with only a single encoder-decoder. (**a**) The predicted and original values of exhaust gas temperature; (**b**) the predicted and original values of core speed; (**c**) the predicted and original values of fuel flow.

Figure 14 shows the mining results of the mapping relationship mining model with only a single encoder-decoder. The time series of parameters except for EGT, FF, and CS is input to the encoder. The decoder inputs the encoding and the time series at time 1 to time *T*-1 of EGT, FF, and CS. The decoder outputs the time series at time *T* of EGT, FF, and CS. Although this model can also reflect the trend of gas-path changes, the mapping relationship is not sufficiently accurate and has a large error, which proves the superiority of the model structure proposed in this paper.

Table 6 shows the scores of these two models. Their mapping relationship mining errors are too large, the mined mapping relationships are of low quality, and the extracted features cannot be used for anomaly detection. To save space, the feature extraction results of these two models are not presented.

**Figure 15:**
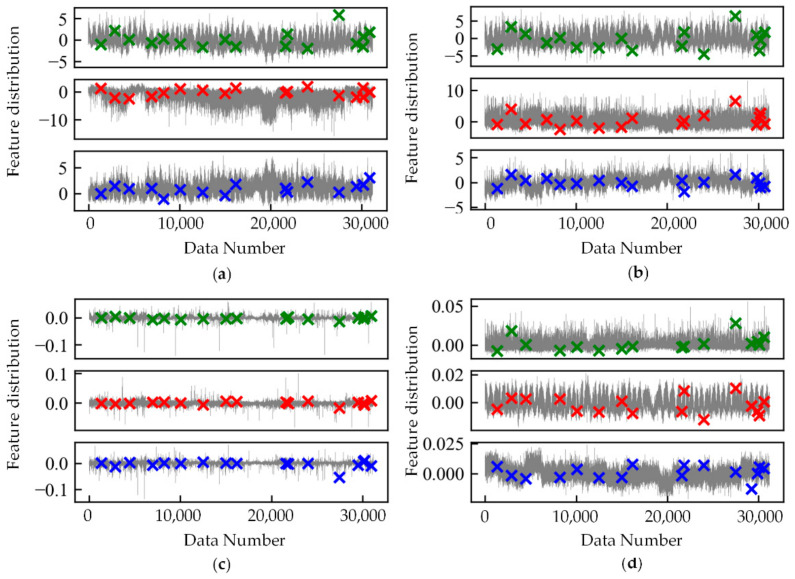
Results of other feature extraction methods. (**a**) Feature extraction results of PCA; (**b**) feature extraction results of AE; (**c**) feature extraction result of ICA; (**d**) feature extraction results of LLE.

Figure 15 shows the feature extraction results of four classical methods. The *x*-axis represents the data number, the *y*-axis represents the extracted features (without clear physical meaning and dimensionality), and the points marked as “×” represent known anomalies. The feature extraction results of these four methods can hardly show apparent anomalies, which proves the superiority of the model in this paper. Anomaly detection results of these methods are given in Section 5.5.

### 5.4. Result of Anomaly Detection

The extracted features of different engines vary greatly, Separate anomaly detection for each engine can effectively improve the accuracy. Table 7 shows the anomaly detection results of the method proposed in this paper (WSFE + IDPC). Weakly supervised anomaly detection (weakly supervised IDPC) was used for some engines with more than one anomaly sample and unsupervised anomaly detection (unsupervised IDPC) was used for some engines with only one anomaly sample. The old engines used the WSFE model trained with new engine data to make the anomalies more apparent. The other engines used the WSFE model trained with mixed engine data. In the weakly supervised IDPC, it is necessary to randomly delete some anomaly labels, use the remaining label for weakly supervised IDPC, and use the deleted labels for verifying the accuracy of anomaly detection. In the unsupervised IDPC, it is necessary to adjust the clustering parameters according to the four evaluation indexes mentioned above, use the clustering with the highest score for anomaly detection, and use the labels to verifying the accuracy. The method proposed in this paper can find most of the anomalies with few false alarms, which meets the practical application requirements.

### 5.5. Comparative Experiments for Anomaly Detection

To verify the superiority of the proposed method, anomaly detection results of multiple models and the state-of-the-art methods were used as comparative tests. Table 8 shows the anomaly detection results of different models, the underlined values indicate that the anomaly detection method was weakly supervised. The model training method and accuracy verification method are the same as those in Section 5.4. To save space, some results of ineffective models are not shown in detail. Table 9 shows the anomaly detection results of the state-of-the-art algorithms; their source code was obtained from Pyod [41].

In model 1, the feature extraction method without $Loss_{WSCE}$ and classifier was combined with IDPC for anomaly detection. In model 2, the feature extraction method without $Loss_{WSCE}$ and classifier was combined with the DPC for anomaly detection. In model 3, the WSFE is combined with the DPC for anomaly detection. In model 4, the WSFE is combined with IDPC without the new threshold for anomaly detection; In model 5, WSFE is combined with OPTICS for anomaly detection. In model 6, LLE is combined with IDPC for anomaly detection. In model 7, AE is combined with DPC for anomaly detection. In model 8, PCA is combined with IDPC without the new threshold for anomaly detection. In model 9, LLE is combined with the DPC for anomaly detection. In model 10, PCA is combined with OPTICS for anomaly detection. In models 1 to 5, the old engines use the WSFE model trained with new engine data and the other engines use the WSFE model trained with mixed engine data.

According to Table 8, the features extracted by traditional feature extraction methods cannot accurately reflect the characteristics of anomalies. Regardless of what anomaly detection methods are used, anomalies cannot be effectively detected. By adding special structures, the feature extraction method in this paper (WSFE) could better reflect the characteristics of anomalies, and most of the anomalies could be detected by different anomaly detection methods. The anomaly detection method in this paper (WSFE + IDPC) could detect most of the known anomalies and had the fewest false alarms. IDPC performed better especially in weakly supervised conditions, which proves the superiority of the WSFE, the $Loss_{WSCE}$, IDPC, and the weakly supervised clustering parameter adjustment strategy.

According to Table 9, anomaly detection algorithms which perform well in other fields cannot be directly applied to the task of gas-path anomaly detection. Even if the anomaly threshold of these algorithms is set to 10%, they still have a very high false negative rate, which proves the superiority of the proposed method.

## 6. Discussion

The purpose of this study was to realize an accurate and reliable gas-path anomaly detection method for civil aero-engines. A weakly supervised feature extraction method based on mapping relation mining and an anomaly detection method based on improved density peak clustering were proposed. The mapping relationship mining model consisting of the LSTM and the attention mechanism could accurately mine the mapping relationship between the gas-path parameters. By combining a classifier and a new loss function, named weakly supervised cross-entropy under extreme class imbalance, the learned mapping relationship was strongly forced to distinguish anomaly samples from normal samples. The deviation degree between the true values and predicted value of the mapping relationship could be used as features to improve the accuracy of the anomaly detection, which had good interpretability. The anomaly detection algorithm based on improved density peak clustering could detect anomalies accurately with fewer false alarms. The weakly supervised clustering parameter adjustment strategy could reduce the parameter adjustment complexity by using weakly supervised labels. Finally, the experimental results showed that the method proposed in this paper is a gas-path anomaly detection method with wide application prospects.

The feature extraction experiments showed that the feature extraction method could intuitively reflect most of the anomalies, and most of the anomalies were very different from the predicted values derived from the mapping relationship, which is significantly better than traditional feature extraction methods. The anomaly detection experiments showed that the anomaly detection method proposed in this paper had a better ability to identify anomaly features, and the new outlier threshold had a better ability to capture outliers. Excluding outliers from the clustering process can significantly reduce the impact of outliers on clustering results. However, different K-nearest neighbor numbers had a certain influence on clustering results, and they had to be adjusted several times, which was subjective to some extent. Therefore, a weakly supervised clustering adjustment strategy was proposed, and anomaly labels were used to guide the parameter adjustment process, which greatly reduced the difficulty of parameter adjustment. However, this adjustment strategy was only applicable to engines with more than one anomaly sample, which limits the strategy application.

It is worth noting that the features extracted in this paper can reflect the health status of different engines. Some normal samples of new engines are very stable, while some normal samples of old engines show an obvious deterioration trend. Models trained with different training datasets have different abilities to reflect anomalies, and models trained with new engine data are more sensitive to anomalies. These extracted features of different engines must be detected separately to avoid reducing the anomaly detection accuracy greatly. It can be speculated that the dataset used in this paper was not sufficient and the model was not trained by enough engines with different health statuses, which led to an insufficient generalization ability for engines with poor health status and obvious differences in the features of different engines.

In view of the limitations of the model proposed in this paper, more original gas-path parameters and more anomaly samples should be gathered in the future to improve the generalization ability of the feature extraction model and to avoid detecting anomalies for each engine alone, which could improve the performance of the model in engineering applications. If more anomaly samples can be collected, the anomaly detection method will be further verified and optimized.

## 7. Conclusions

In this paper, a weakly supervised anomaly detection method based on mapping relationship mining and improved density peak clustering was proposed. First, a mapping relationship mining model for gas-path parameters was established using the attention mechanism and LSTM, and the deviation degree between the predicted and original values was used as the feature for anomaly detection. The weakly supervised cross-entropy loss function under extreme class imbalance combined with a classifier was proposed to force the mapping relationship mining model to reflect the difference between normal samples and anomaly samples. Finally, the improved density peak clustering-based anomaly detection method and the clustering adjustment strategy under weakly supervised conditions were proposed to realize an anomaly detection method without a complex parameter adjustment process. Compared with traditional methods, the feature extraction method proposed in this paper could better reflect the performance change of the engines, and the anomalous samples were more obvious. The anomaly detection algorithm proposed in this paper could detect anomalies more accurately with minor false alarms.

## 8. Limitations and Directions for Future Research

It is important to note that the method proposed in this paper has some limitations with regard to engineering applications. As mentioned above, the method in this paper needs sufficient samples, otherwise there will be a big difference between the models trained by different training sets. The method proposed in this paper can have a good effect when the proportion of anomalies is relatively small, but it still needs enough anomaly samples. The dataset used in this paper had only 17 anomaly labels, which cannot fully reflect the superiority of the method. To improve the accuracy, anomaly detection should be carried out separately for each engine, which is not conducive to the application. Most importantly, the dataset used in this paper had incomplete labels, so it was difficult to determine whether the unlabeled data was normal or not, and the accuracy of anomaly detection results was difficult to evaluate. To reduce the false negatives, some false positives had to be tolerated.

In the future, a sufficient dataset could be collected to train the model proposed in this paper. With more anomaly samples, the weakly supervised cross-entropy will be further verified and optimized, and how to directly realize anomaly detection under extreme sample imbalance using the classifier can be researched. Based on the feature extraction method in this paper, a secondary feature extraction method based on time-domain features, such as slope, amplitude, kurtosis, margin, etc., could be researched to unify anomaly detection for all engines. If a supervised dataset can be obtained, it will be more conducive to adjusting the model and evaluating the results.

## Figures and Tables

**Table 1:** Anomaly numbers for each engine.

Engine number	643787	699184	699833	569172	569469	645580	645764	697511	699244	699501	699796	699896
Known anomalies	2	4	2	1	1	1	1	1	1	1	1	1

**Table 2:** Original gas-path parameters of civil aero-engines.

Sample	*x*_1_	*x*_2_	⋯	*x*_1329_	⋯	*x*_31190_
Anomaly?	No	No	⋯	Yes	⋯	No
Alt (Ft)	26,603	32,101	⋯	33,104	⋯	33,104
FS (%)	83.1	87.9	⋯	88.7	⋯	88
CS (%)	93.1	96.2	⋯	96.6	⋯	95.9
TAT (°C)	−13.8	−8.3	⋯	−8	⋯	−10.5
EGT (°C)	609.5	680.1	⋯	686.1	⋯	676
OT (°C)	81	78	⋯	89	⋯	89
OP (MPa)	50	52	⋯	49	⋯	48
Ma (Ma)	0.739	0.769	⋯	0.777	⋯	0.765
FF (P/h)	3441.416	3366.459	⋯	3368.663	⋯	3260.637

**Table 3:** Hyperparameters.

Model	Parameters	Setting
WSFE	Length of time-series	10
	Encoder LSTM unit number	50
	Decoder LSTM unit number	40
	Classifier LSTM unit number	100, 100, 100
	Imbalanced coefficient *α*	3000
	Focusing coefficient *γ*	5
	Adjustment coefficient *β*	1.5
	FC layer unit quantity FC layer activation function	1
		sigmoid
	Learning rate	0.001
	Batch size	1024
	Epochs	1000
	Iteration stop condition	The loss change rate of the test set is less than 0.01%
IDPC	Initial K-nearest neighbor number	0.9 * total sample numbers
	K-nearest neighbor number of iteration stop	1
	Initial minority class sample number	0.01 * total sample numbers
	Minority class sample increasing number per iteration	0.01 * total sample numbers
	Minority class sample number when iteration stops	0.1 * total sample numbers

*: Multiplication.

**Table 4:** Scores for mining mapping relationships under different training methods.

Evaluation Indicator	Mixed Engine Data	New Engine Data	Old Engine Data
RMSE	0.099	0.133	0.142
MAE	0.076	0.100	0.113
MAPE	38.096	47.139	58.993
R^2^	0.990	0.982	0.979

**Table 5:** Scores of the feature extraction model without $Loss_{WSCE}$ and classifier.

Evaluation Indicator	Mixed Engine Data	New Engine Data	Old Engine Data	The Method Proposed in this Paper
RMSE	0.099	0.135	0.138	0.099
MAE	0.078	0.101	0.129	0.076
MAPE	37. 960	49.001	57.996	38.096
R^2^	0.993	0.980	0.979	0.990

**Table 6:** Scores of the two models.

Evaluation Indicator	Only Encoder-Decoder and LSTM	Only One Encoder-Decoder	The Method Proposed in This Paper
RMSE	0.422	0.496	0.099
MAE	0.321	0.333	0.076
MAPE	173.931	93.004	38.096
R^2^	0.821	0.753	0.990

**Table 7:** Anomaly detection result for the WSFE + IDPC.

	Engine Number	Known Anomalies	False Negatives	Total Number of False Alarms /Total Number of Samples
Weakly supervised	643787	2	0	2.404%
	699184	4	1	1.199%
	699833	2	0	0.776%
Unsupervised	569172	1	1	-
	569469	1	0	0.810%
	645580	1	0	9.295%
	645764	1	0	1.350%
	697511	1	0	0.597%
	699244	1	0	1.742%
	699501	1	0	0.611%
	699796	1	1	-
	699896	1	1	-

**Table 8:** Anomaly detection results of different models.

Engine Number	Total Number of False Alarms/Total Number of Samples										
	Model 1	Model 2	Model 3	Model 4	Model 5	Model 6	Model 7	Model 8	Model 9	Model 10	WSFE + IDPC
643787	7.14%	11.36%	4.51%	3.01%	27.13%	-	-	-	-	-	2.404%
699184	1.51%	15.52%	23.46%	28.51%	34.41%	-	-	50.68%	-	-	1.199%
699833	-	15.09%	7.77%	7.91%	26.95%	-	-	-	-	-	0.776%
569172	-	-	-	-	-	-	-	-	-	-	-
569469	4.07%	-	-	-	-	-	21.37%	-	-	-	0.810%
645580	-	9.83%	-	-	-	-	20.14%	-	-	-	9.295%
645764	0.91%	-	-	-	-	-	20.10%	3.11%	9.27%	4.58%	1.350%
697511	0.05%	-	-	-	-	-	-	-	25.33%	-	0.597%
699244	-	-	-	-	-	18.45%	-	-	-	-	1.742%
699501	0.61%	-	-	23.82%	-	-	-	-	24.52%	9.32%	0.611%
699796	-	-	-	-	-	-	32.96%	-	-	-	-
699896	-	-	-	-	-	-	28.71%	-	-	-	-
False negative rate	9/17	12/17	13/17	12/17	13/17	16/17	12/17	15/17	14/17	15/17	4/17

**Table 9:** Anomaly detection results of the state-of-the-art algorithms.

Engine Number	Total Number of False Alarms/Total Number of Samples							
	ABOD	KNN	I-Forest	HBOS	LOF	SOS	LSCP	WSFE + IDPC
569172	-	-	-	-	-	10.01%	-	2.404%
569469	-	-	-	-	-	10.01%	-	1.199%
643787	-	-	-	-	-	-	-	0.776%
645580	-	-	-	-	-	-	-	-
645764	9.88%	8.43%	-	9.29%	8.99%	-	11.00%	0.810%
697511	-	-	-	-	-	-	-	9.295%
699184	-	-	-	-	-	-	-	1.350%
699244	10.14%	8.49%	10.00%	9.64%	9.41%	10.00%	10.00%	0.597%
699501	-	-	10.02%	10.02%	-	-	-	1.742%
699796	10.31%	8.02%	10.01%	9.93%	9.01%	10.01%	10.01%	0.611%
699833	-	-	-	-	-	-	-	-
699896	-	-	-	-	-	-	-	-
False negative rate	14/17	14/17	14/17	13/17	14/17	12/17	14/17	4/17

## Data Availability

Data are included within the article.
